# A Paradigm Shift on the Question of B Cells in Transplantation? Recent Insights on Regulating the Alloresponse

**DOI:** 10.3389/fimmu.2017.00080

**Published:** 2017-02-02

**Authors:** Daniel J. Firl, Gilles Benichou, James I. Kim, Heidi Yeh

**Affiliations:** ^1^Transplant Center, Massachusetts General Hospital, Harvard Medical School, Boston, MA, USA; ^2^Howard Hughes Medical Institute, Chevy Chase, MD, USA

**Keywords:** regulatory B cells, transplant tolerance, antigen presentation, allorecognition, transplant rejection, autoimmune diseases

## Abstract

B lymphocytes contribute to acute and chronic allograft rejection through their production of donor-specific antibodies (DSAs). In addition, B cells present allopeptides bound to self-MHC class II molecules and provide costimulation signals to T cells, which are essential to their activation and differentiation into memory T cells. On the other hand, both in laboratory rodents and patients, the concept of effector T cell regulation by B cells is gaining traction in the field of transplantation. Specifically, clinical trials using anti-CD20 monoclonal antibodies to deplete B cells and reverse DSA had a deleterious effect on rates of acute cellular rejection; a peculiar finding that calls into question a central paradigm in transplantation. Additional work in humans has characterized IL-10-producing B cells (IgM memory and transitional B cells), which suppress the proliferation and inflammatory cytokine productions of effector T cells *in vitro*. Understanding the mechanisms of regulating the alloresponse is critical if we are to achieve operational tolerance across transplantation. This review will focus on recent evidence in murine and human transplantation with respect to non-traditional roles for B cells in determining clinical outcomes.

## Introduction

Allorecognition refers to the detection by the immune system of polymorphic determinants expressed by different individuals of the same species (alloantigens) (1–3). After transplantation of allogeneic organs or tissues, recognition of alloantigens by host leukocytes initiates an inflammatory immune response leading to graft rejection (4, 5). It is now established that certain leukocytes of the innate immune system, including NK cells and macrophages, can distinguish between self- and non-self antigens and thereby contribute to the alloresponse (6–8). However, allorecognition by T lymphocytes of the adaptive immune system is the driving force behind alloimmunity and allograft rejection in vertebrates. After transplantation, graft MHC class II^+^ cells as well as donor-derived extracellular vesicles traffic to the recipient lymphoid organs where they activate CD4^+^ allospecific T cells (9–12). This process occurs *via* two distinct pathways: *direct allorecognition* in which T cells recognize intact donor MHC molecules as well as the *semi-direct mechanism* dependent on donor-derived MHC–peptide complex, which traffics *via* extracellular vesicles to be presented upon recipient antigen-presenting cells (APCs). In this case, the recipient dendritic cell (DC) becomes chimeric for donor allopeptide–MHC complex and can present to donor responsive CD8^+^ T cells through the direct pathway (13). It is important to note that some complex can undergo internalization, degradation, loading, and presentation on MHC-II to CD4^+^ T cells in the same manner as below in the indirect pathway. Thus, a single DC can present to both CD4^+^ and CD8^+^ cells resulting in a linked activation of T cells (14). The *indirect pathway* involves T cells, which interact with donor peptides bound to recipient MHC molecules on host APCs (15–18). This process leads to the differentiation of CD8^+^ cytotoxic T lymphocytes (CTL) and to plasmocytes (B cells), which produce donor-specific antibodies (DSAs) (19). B cells play a key role in acute and chronic allograft rejection through their production of DSAs, a process requiring help from CD4^+^ T cells activated indirectly (20). In addition, B cells serve as APCs and present alloantigen peptides to T cells thereby contributing to their activation and differentiation into memory T cells (21, 22). On the other hand, certain B cell subsets can suppress inflammatory alloreactive T cells and promote allograft tolerance (23–27). In this article, we present recent data from human and animal studies that raise exciting new possibilities for B cells in antigen presentation and T cell regulation relevant to transplantation.

## Allorecognition by B Cells

B cells have a critical role in indirect allorecognition. The traditional immunological concepts for developing an adaptive response to any given protein antigen underpin the so-called indirect pathway of allorecognition. Recipient T cells recognize processed allopeptide–self-MHC-II complexes on recipient APCs (28–30). The indirect response is primarily CD4^+^ T cell-driven due to the involvement of self-MHC-II molecules (31, 32). Following recognition of cognate antigen on DCs in the T cell zone, these CD4^+^ T cells upregulate BCL6, CXCR5, and CD40L and downregulate CCR7, which allows them to migrate to the follicle where they take on the follicular T helper cell phenotype (33). These cells can then instruct follicular B cells, which have internalized donor antigen to seed germinal centers (GCs) *via* the CD40L/CD40 axis as well as the secretion of IL-21 promoting the differentiation of CD40L stimulated B cells (34). These B cells undergo somatic hypermutation, a critical step to generating high-affinity DSA (35). They also class switch and some differentiate into plasma cells (with highest BCR signal strength) or memory B cells if density and tonicity of the B cell receptor signaling are insufficient to differentiate to a plasma or GC B cell (36). Thus, the presence of DSA can be used as a proxy measure of the activity of the indirect pathway (37, 38). In addition to alloreactive or DSA, B cells can generate antibody responses against non-HLA self-peptides, the angiotensin II receptor is an example of an activating antibody leading to a functional change following renal transplantation (39). The extent to which these antibodies contribute to rejection, especially chronic vascular type rejection is as of yet unclear; however, the mechanism of generation in the face of varying degrees of allograft tolerance (DSA levels) is intriguing (40).

## B Cells as APCs

B cells are likely to play a role in antigen presentation associated with indirect activation of donor-specific T cells. For example, the presence of CD20^+^ cells in renal allografts is associated with poor outcomes and acute cellular rejection, but not necessarily antibody-mediated rejection (AMR), in renal transplantation (41). B cells present in these grafts presumably mediate their effects through alloantigen presentation and ICOS/CD28 costimulation of T cells leading to their activation and expansion (42). Graft infiltrating CD20^+^CD27^+^ memory B cells survey for cognate antigen prior to expanding and seeding GCs, a process leading to increased DSA production and subsequent acute and chronic rejection (43). These DSAs have the potential to greatly modify the interplay of donor antigen and recipient tolerance since bound antibodies have the potential to fix complement and lead to increased tissue damage and increased antigen presentation, as well as epitope spreading, leading to tissue-specific responses as in the indirect pathway described above (44).

## Role of B Cells in Suppressing Inflammatory Alloimmunity

B cells may not always act as pro-inflammatory players. In human renal transplantation, B cells were recently shown to have a regulatory role on T cell alloresponses *in vitro* using peripheral blood from 65 patients with biopsy-proven AMR, non-immune related graft dysfunction, or stable graft function (45). The authors found many biopsy-proven AMR samples that did not demonstrate an anti-donor IFN-gamma response unless CD25^+^ (regulatory T cells) and CD19^+^ cells (B cells) were depleted. More importantly, depletion of these cells also restored alloresponsiveness in patients with no histological signs of immune-mediated graft dysfunction. Alloresponsiveness was dependent on B–T interactions (with CD19^+^ cells acting as APCs *in vitro*).

A clinical trial in renal transplantation compared the efficacy of rituximab, a monoclonal anti-CD20 antibody, with daclizumab, a monoclonal anti-CD25 antibody (46) as induction therapy. This trial was halted early due to dramatically increased rates of biopsy-confirmed acute rejection (within the first 3 months post-transplant) in the rituximab-treated group compared with daclizumab (83 versus 14%; *p* = 0.01). In fact, the rate of acute rejection observed in the rituximab-treated group exceeded previously observed rates in recipients that did not receive any induction therapy (~35%), suggesting that B cell depletion actually increased alloreactivity. Another study sought to evaluate rituximab for desensitization prior to HLA-incompatible live donor renal transplantation. Rituximab-treated recipients exhibited a trend toward higher rates of acute rejection and greater number of episodes of rejection compared with non-rituximab recipients (47). These studies’ results are in line with animal models showing worsening of disease severity along several T-dependent autoimmune models including ulcerative colitis (48), psoriasis (49), and autoimmune encephalomyelitis/multiple sclerosis (EAE/MS) (50) following anti-CD20 mAb-mediated B cell depletion, despite decreases in circulating autoantibodies, underscoring the antibody-independent role of B cells in autoimmunity. However, other studies including rituximab in the induction period for ABO incompatible desensitization did not show statistically significant differences in rates of acute rejection, although they did raise the concern of possible increased risk of cardiac mortality following B cell depletion (51, 52).

The role of B cells with regulatory potential has also been explored in human hematopoietic stem cell transplantation. Chronic graft versus host disease (cGVHD) is a debilitating complication that carries a poor prognosis in patients who fail to respond to corticosteroids (53, 54). A frequent observation in GVHD is increased titers of autoantibody that demonstrates a loss of peripheral B cell tolerance (54). Khoder et al. examined the frequencies of regulatory B cells in GVHD and healthy controls and found that the ratio of IL-10^+^ B cells to IFN-gamma CD4^+^ T cells was greatly reduced in cGVHD patients compared to stable controls (55). They found B cells with regulatory function (Bregs) (as measured by the ability to suppress CD4^+^ T cell proliferation and effector function *in vitro*) in both the IgM memory (CD19^+^IgM^+^CD27^+^) and transitional B cell (TrB; CD19^+^CD24^hi^CD38^hi^) compartments. They also demonstrated that the regulatory potential of these cells required cell–cell contact by coculturing both IgM memory and TrB cells in transwell plates with anti-CD3 and anti-CD28 antibody-activated CD4^+^ T cells. CD80/CD86 blockade in coculture systems was also found to be deleterious to the development of full regulatory effect by Bregs, and that this effect was independent of CD80/PD-1 interactions. The necessity for cell–cell contact combined with the ability of B cells to act as APC raises the question of whether Bregs are antigen-specific *via* either the B cell receptor or MHC, although there have been no reports of direct evidence supporting either possibility.

Future work needs to be done to clarify the ontogeny of donor-specific “regulatory” B cells [current definitions rely on functional production of IL-10 (56–58)]. The regulatory B cell populations in murine models are more fully characterized and reliably defined by phenotypic markers compared with humans. Although no fewer than 10 subsets have been defined as “Bregs,” most work has been done on either marginal zone precursor B2 cells or B10 cells, which are typically CD19^+^CD1d^hi^CD5^+^ (a population, which overlaps with marginal zone B2 cells, marginal zone precursor B2 cells, and B1 cells) (59). However, many still perform *in vitro* assays using anti-CD40 antibodies, and PMA-ionomycin, followed by monensin or brefeldin treatment to stimulate IL-10-competent B cells to produce and retain this cytokine for intracellular staining (25). In humans, only a small percentage of cells identified as potentially regulatory by phenotypic markers produce IL-10, a finding that makes translation more difficult (60, 61).

One of the first animal models to demonstrate the regulatory role of B cells in transplantation was performed in a murine renal transplantation model where greater efficiency of tolerogenesis was observed by transplanting donor B cells at the time of renal transplantation than with donor T cells (62). Since that time, laboratory efforts have identified several subtypes of B cells with regulatory potential (63).

In a murine model of pancreatic islet allotransplantation, T cell Ig domain and mucin domain protein 1 (TIM-1), a costimulatory molecule was shown to modulate CD4^+^ T cell reactivity and serves as a marker of Bregs (27). TIM-1 broadly marked Bregs with significant overlap with IL-10^+^ capable cells. In fact, TIM-1 ligation actually enhanced production and secretion of IL-4 and IL-10 by B cells. Compared to other reports, this group was able to more reliably identify IL-10^+^ cells in peripheral tissues and secondary lymphoid organs as compared to spleen using TIM-1 positivity as opposed to a non-specific CD19^+^CD1d^hi^CD5^+^ gate. Finally, they were able to promote tolerogenesis *via* RMT1-10, an anti-TIM-1 mAb, which simulates CD4^+^ binding. This work was furthered by identifying the role of Breg-derived TGF-beta in inducing Tregs and in promoting tolerance to fully MHC-mismatched pancreatic islet transplants. Tolerance induction in these mice was transferrable through injection of naïve mice with B cells from dual antibody-treated recipients (anti-CD45RB and anti-TIM-1) (24). This dual therapy promoted TGF-beta secretion by TIM-1^+^ B cells and led to a substantial increase in Treg frequencies, which was blocked by anti-TGF-beta antibody (26).

## Conclusion

It is clear that great strides are being made across the field of transplantation with respect to the understanding of the many roles of B cells. B cells are unique in their ability to produce antibodies, which can kill donor cells *via* antibody-dependent cell-mediated cytotoxicity and complement fixation. In addition, B cells are efficient APCs providing help to T cells thereby polarizing the T cell response and promoting the differentiation of memory T cells. However, mechanistically informed clinical trials, which sought to take advantage of the indirect pathway of allorecognition *via* CD20^+^ antibody treatment to deplete recipient B cells, resulted in increased rates of acute cellular rejection. This peculiar result challenges the single faceted view of B cells as solely pro-inflammatory and supports the human relevance of recent laboratory work in rodents, which has demonstrated immunoregulatory roles for several B cell subsets. Future work needs to characterize the transcriptome of Bregs in an effort to identify a transcription factor necessary for function regulation such as Foxp3 in Tregs. Critical questions remain about whether the variety of reported Bregs are indeed separate cell subsets or merely different activation states of B cells across development. This would help to explain such diverse findings in B10, marginal zone precursors, and TIM-1^+^ B cells and would open up the exploration of what cytokine environment polarizes a Breg and might be useful in clinical transplantation.

## Author Contributions

All authors listed have made substantial, direct, and intellectual contribution to the work and approved it for publication.

## Conflict of Interest Statement

The authors declare that the research was conducted in the absence of any commercial or financial relationships that could be construed as a potential conflict of interest.

## Abbreviations

AMR, antibody-mediated rejection; APC, antigen-presenting cell; Breg, B regulatory cells; cGVHD, chronic graft versus host disease; CTL, cytotoxic T lymphocytes; DSAs, donor-specific antibodies; GC, germinal center; HSCT, hematopoietic stem cell transplant; TrB, transitional B cells.
